# Impact of Orientation on the Vitamin D Weighted Exposure of a Human in an Urban Environment

**DOI:** 10.3390/ijerph14080920

**Published:** 2017-08-16

**Authors:** Michael Schrempf, Nadine Thuns, Kezia Lange, Gunther Seckmeyer

**Affiliations:** Institute of Meteorology and Climatology, Leibniz Universität Hannover, 30419 Hannover, Germany; thuns@muk.uni-hannover.de (N.T.); lange@muk.uni-hannover.de (K.L.); seckmeyer@muk.uni-hannover.de (G.S.)

**Keywords:** UV radiation, human exposure, vitamin D, urban environment, hemispherical sky images, radiance

## Abstract

The vitamin D_3_-weighted UV exposure of a human with vertical posture was calculated for urban locations to investigate the impact of orientation and obstructions on the exposure. Human exposure was calculated by using the 3D geometry of a human and integrating the radiance, i.e., the radiant energy from the direct solar beam and the diffuse sky radiation from different incident and azimuth angles. Obstructions of the sky are derived from hemispherical images, which are recorded by a digital camera with a fisheye lens. Due to the low reflectivity of most surfaces in the UV range, the radiance from obstructed sky regions was neglected. For spring equinox (21 March), the exposure of a human model with winter clothing in an environment where obstructions cover 40% of the sky varies by up to 25%, depending on the orientation of the human model to the sun. The calculation of the accumulated vitamin D_3_-weighted exposure of a human with winter clothing walking during lunch break shows that human exposure is reduced by the obstruction of buildings and vegetation by 40%.

## 1. Introduction

Ultraviolet radiation from the sun causes a considerable global disease burden, including acute and chronic health effects on the skin, the eyes and the immune system [1]. Negative effects of an increased UV exposure include, for example, erythema, sunburn and keratitis [2,3]. In addition, UV radiation is a fundamental parameter in the genesis of skin cancer [4,5]. On the other hand, UV is essential for the vitamin D_3_ production of humans [1,6]. In the following, vitamin D is used as a general term, whereas we use the expression vitamin D_3_ to describe UV-related issues. There is evidence that suggests vitamin D levels could be seen as an indicator of health risk relating to some sorts of cancers, infectious diseases (e.g., dental caries, pneumonia), and autoimmune diseases (e.g., diabetes mellitus type 1, multiple sclerosis) among others. Furthermore, there are established links with musculoskeletal health, Parkinson’s disease and rickets [7,8]. Evidence is abundant that UVB exposure, vitamin D intake, and vitamin D concentrations are inversely correlated with many cancers (e.g., breast, lung, ovarian and colorectal cancer) [9,10,11]. The main source of vitamin D for humans is the vitamin D synthesis in the human skin due to solar UVB radiation (280–315 nm), although radiation between 280 and 290 nm is almost completely absorbed by ozone in the atmosphere. Dietary intake contributes only a small percentage (10%) to the necessary supply [12]. There are large seasonal differences in the production of vitamin D [13,14], which are mainly caused by the varying solar zenith angle and the different areas of skin that are exposed to solar radiation. Although vitamin D_3_ can be stored in body fat and mobilized during winter months when little, if any, vitamin D_3_ is produced in the skin [15], more than 50% of the German population have an insufficient vitamin D supply [16]. In this context, several studies refer to the so-called “vitamin D winter”, which is the period of time where an adequate vitamin D status cannot be gained by solar UV exposure. The vitamin D winter for mid-northern latitudes is often stated to range from October to March [13,17,18,19]. Insufficient vitamin D levels can not only occur in winter time because of low UV exposure values, but also due to environmental obstructions (e.g., vegetation or buildings). The calculation of the vitamin D_3_-weighted human exposure in an urban environment is necessary in order to estimate if sufficient vitamin D_3_ could be synthesized in everyday situations.

## 2. Materials and Methods

### 2.1. State of the Art

**Figure 1:**
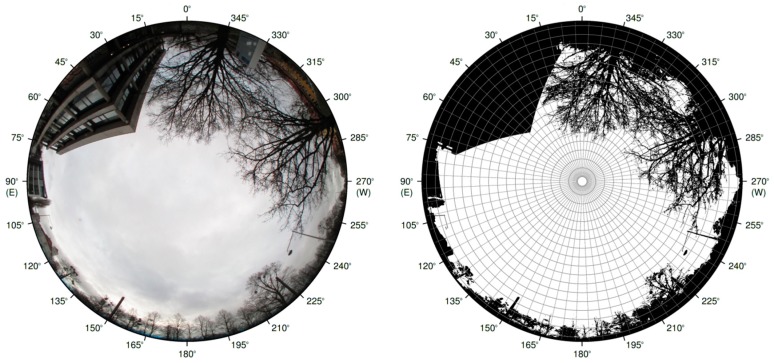
Hemispherical image taken in front of the Institute of Meteorology and Climatology of the Leibniz Universität Hannover (**left**) and the image with the derived obstruction information (**right**), where the derived obstructions shadowing the sky are shown in black and sky in white. The image is segmented by the grid shown, which illustrates the different directions (sky segments). The ratio of the amount of pixels characterized as obstructions and the total pixel count results in 39% obstructed sky.

Earlier exposure investigations (e.g., by Diffey [17] and McKenzie et al. [20]) were based on the irradiance incident on horizontal or vertical surfaces. To better represent the UV dose of a human outdoors, Godar and Pope [21,22,23] converted the weighted irradiance of a horizontal plane into that of a cylinder by using geometric conversion factors. However, these theoretical estimations were performed for an unobstructed location only. Kawanishi [24] used hemispherical images to estimate the shadowing of the sky by sunscreens, although the subject of his study was the protection against erythemally-weighted radiation on a horizontal plane. Parisi et al. [25] included the shadowing of the sky by trees in their study of UV exposure. For their investigations, they used UV dosimeters on rotating mannequins that matched the stature of an average human. The instruments were distributed over the entire mannequin surface to measure the radiation on as much of the body surface area as possible. In a recent study, Parisi et al. [26] attached dosimeters at the vertex and forehead of a mannequin head and compared measurements conducted under different shade structures and in direct sun. However, rather than vitamin D_3_-weighted human exposure, erythemally weighted doses were being investigated in these studies. Additionally, one single location may not be representative for an urban environment, because obstructions at different locations differ in shape and size, and therefore cover different parts of the sky. The irradiance on a horizontal surface should not be used for exposure calculations because the radiation field of the sky would have to be described by a single number only, which does not reflect the complex reality. Instead, the quantity “radiance” should be used (describing the radiant energy per unit solid angle and per unit area), thus taking into account the complex radiation field. Seckmeyer et al. [27] developed an exposure model based on radiance in combination with a 3D-voxel model of a human. Most surfaces show a very low reflectivity in the UV range, therefore the radiance from obstructed directions can be neglected, which enables the determination of the exposure of a human by calculating the multidirectional downwelling radiance originating from unobstructed directions. The obstructions of a location in an urban environment can, for example, be seen in Figure 1.

We refer to the method of Seckmeyer et al. [27] to calculate biologically-weighted human exposure by integrating the incident solar spectral radiance over all relevant parts of the human body. The area elements of the human model are defined as projection area $A_{\mathrm{proj}}$, which takes into account the geometrical properties as well as the clothing of the human.

We define the human exposure as the total biologically-weighted radiant energy received by the exposed body surfaces of a human. The equation for the calculation of the human exposure is given by:

(1) $$Human\;exposure_{\mathrm{VitD}}=\int_{t_{1}}^{t_{2}}\int_{0}^{\pi /2\;}\int_{0}^{2\pi \;}\;L_{\mathrm{VitD}}\left(\varepsilon ,\varphi ,t\right)\cdot \;A_{\mathrm{proj}}\left(\varepsilon ,\varphi \right)\;d\varphi \;d\varepsilon \;dt.$$

where $L_{\mathrm{VitD}}$ is defined as the vitamin D_3_-weighted radiance and the different directions in the sky are defined by the incident angle (ε) and the azimuth angle (φ). It should be noted that the quantity “human exposure”, defined in Equation (1), must not be confused with the quantity “radiant exposure”, which is defined by the CIE as the time integral of the irradiance [28]. In the following, the expression exposure always relates to the human exposure defined in this study.

**Figure 2:**
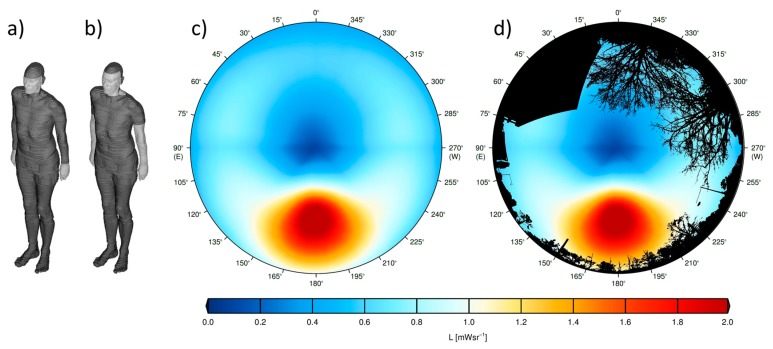
Visualization of the front of the model human with winter clothing (**a**) and summer clothing (**b**); The vitamin D_3_-weighted radiance on 21 March at noon, weighted with the geometry of a human with winter clothing oriented south (180°) is shown in (**c**); The combination of the weighted radiance distribution from (**c**) and the obstruction information from Figure 1 is shown in (**d**).

The human model used in this study is based on a computed tomography scan of a patient, who is 38 years old, 176 cm of height, has a weight of 68.9 kg and skin type 2, and thus approximately represents an average male adult [27,29]. The model can wear winter clothing, where only face and hands are exposed (1455 cm^2^ exposed skin area, 93% covered skin area to the total skin area) and also summer clothing, where face, neck, arms and hands are exposed (4160 cm^2^ exposed skin area, 80% covered skin area to the total skin area). In Figure 2, the visualized voxel models wear winter and summer clothing, and are shown as front perspective from the direction φ = 150 and ε = 30.

### 2.2. Advanced Model

The obstruction of the sky is calculated using hemispherical images taken by a digital camera with a fisheye lens (see, for example, Figure 1). The projection function of the fisheye lens is equidistant, which provides proper segmentation in equal solid angles [30]. For the derivation of the obstruction, a threshold with values between 0 and 255 is introduced, which is calculated as the mean of the count values of the red, green and blue color of each pixel, and can be expressed as:

(2) $$threshold_{i,j}=\;\frac{red_{i,j}\;+\;green_{i,j}\;+\;blue_{i,j}}{3}$$

Pixels with a calculated threshold lower than 125 are defined as obstructions, the others are identified as unobstructed sky. Obstructions can best be detected in hemispherical images taken under overcast conditions, since the obstructions are relatively dark compared to the sky. The albedo of typical surfaces (e.g., street, concrete, vegetation) in the UVB wavelength region is small, with values between 0.02 and 0.1 [31,32]. Seckmeyer et al. [27] stated that for an albedo of 0.02, the extra exposure from an isotropic surface reflection is lower than 3% and can therefore be neglected for low albedo values. For this investigation, we assume that the reflectivity of the surface materials of the detected obstructions is low and can be neglected. It should be noted that this assumption may not be appropriate for some surfaces like snow or glass windows. However, snow wasn’t present in our investigations and windows usually do not cover a large fraction of the sky.

Since the spatial resolution of the hemispherical images with about 3 million pixels is much larger than the spatial resolution of the different sky directions used in the exposure model (sky segments, see Figure 1), mean values of the high resolution obstruction information for each sky segment are calculated.

The obstructions derived from selected locations are implemented in the exposure model by extending the method of Seckmeyer et al. [27] by multiplying the obstruction information with the vitamin D_3_-weighted radiance and the geometric factor of each direction. The formula for the calculation of the human exposure weighted with the obstruction information can be expressed as:

(3) $$Human\;exposure_{\mathrm{VitD}}=\int_{t_{1}}^{t_{2}}\int_{0}^{\pi /2\;}\int_{0}^{2\pi \;}\;L_{\mathrm{VitD}}\left(\varepsilon ,\varphi ,t\right)\;\cdot \;obstruction\left(\varepsilon ,\varphi \right)\;\cdot \;A_{\mathrm{proj}}\left(\varepsilon ,\varphi \right)\;d\varphi \;d\varepsilon \;dt.$$

For this study, the simulations were calculated by the DISORT code of the UVSPEC model in the LibRadTran package [33]. As input parameters, a total ozone column of 300 DU, a horizontal visibility of 50 km and an UV albedo of 0.02 were used. In Figure 2, the major contribution to the human exposure originating from areas around the solar disk can be recognized. It should be noted that these areas are still diffuse sky radiation; the solar disk that is the cause for direct irradiance has a much smaller area.

## 3. Results

**Figure 3:**
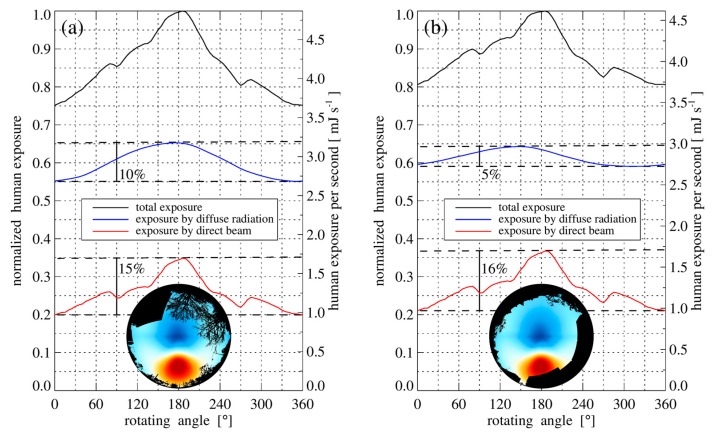
The vitamin D_3-_weighted exposure of a human with winter clothing on 21 March at noon in front of the Institute of Meteorology and Climatology (**a**) and the university cafeteria (**b**) in normalized and absolute units. The human exposure depends on the orientation of the human model towards the sun. The total exposure (black line) is 25% (**a**) and 20% (**b**) smaller if the human is orientated with the face opposite to the sun (0°). The variation of the exposure by the diffuse part of the solar radiation of (**a**) is greater than of (**b**) due to a different distribution of the obstructions. The weighted radiance and the obstruction information of the locations are shown at the bottom of the plots (see also Equation (3) and Figure 2).

In Seckmeyer et al. [27], it was shown that the impact of the orientation of an unclothed human on an obstruction free plane on 21 June at noon is less then ±4% and is primarily caused by the direct part of the solar radiation. In this study, we investigated the dependency of the orientation of a human with winter clothing in an urban environment. The human exposure on 21 March at noon (solar zenith angle of 52°) in front of the Institute of Meteorology and Climatology and in front of the university cafeteria strongly depends on the orientation, and is shown in Figure 3.

**Figure 4:**
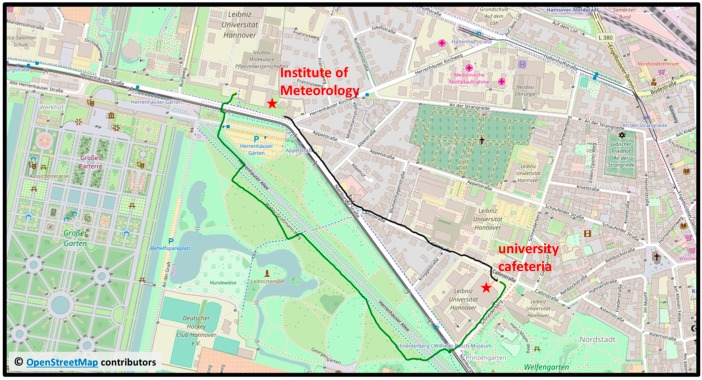
Visualized GPS-data of the two different routes used for the exposure calculation of a moving human in an urban environment. The routes start at the Institute of Meteorology and Climatology and end at the university cafeteria and follow paths through built-up areas (black line) and a recreational park (green line). Map data © OpenStreetMap contributors, CC BY-SA [34].

To mimic an everyday situation for students, the vitamin D_3_-weighted exposure of a moving human was calculated for a winter and a summer scenario (see Table 1). The human is walking on 21 March and 21 June around noon on two different routes from the Institute of Meteorology and Climatology to the university cafeteria. The first route follows the streets “Nienburger Straße” and “Callinstraße” through built-up areas to the cafeteria and takes 10 min by foot. The second route follows a path through the “Georgengarten” and the street “Schneiderberg”, takes 18 min and leads mainly through a recreational park (see also Figure 4). On both routes the human walked on the sunny side of the street to maximize the exposure to solar radiation. The obstruction information was derived in high spatial resolution by moving along the selected footpaths with a GPS-receiver with a sampling rate of 1 s and simultaneously taking hemispherical images with a sampling rate of 4 s. For each image the obstruction was calculated and the related geographic coordinates were assigned. The obstruction information were derived for winter conditions (without foliage) and summer conditions (with foliage).

**Figure 5:**
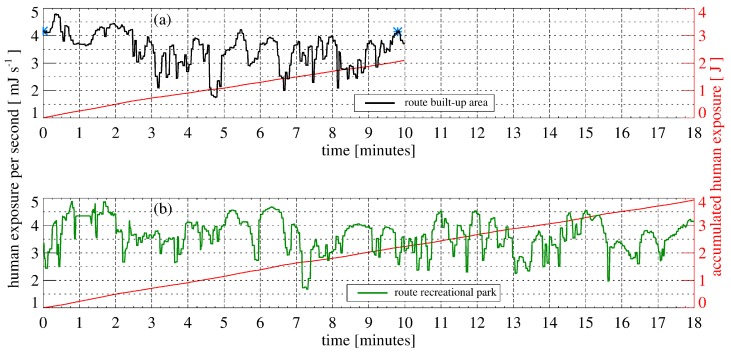
The calculated vitamin D_3_-weighted exposure of a human with winter clothing walking to the university cafeteria on the two selected routes, through a built-up area (**a**) and through a recreational park (**b**), on 21 March is shown as a function of the time. Additionally, the accumulated exposure is displayed in red. The blue stars mark the locations shown in Figure 3.

To calculate the exposure of a moving human the obstruction information nearest to each coordinate of the GPS-receiver was selected and the actual vitamin D_3_-weighted exposure was calculated according to Equation (3). The orientation of the human is necessary for the calculation, and is assumed to be the same as the direction of movement derived from the GPS-receiver data. The calculated exposure of a human with winter clothing walking to the university cafeteria on the two selected footpaths on 21 March is shown as a function of the time in Figure 5. The vitamin D_3_-weighted human exposure on the first route (“Nienburger Straße”) results in 2.1 J, and the human exposure on this route without obstructions would be 3.48 J. Therefore, the exposure is reduced to 2.1 J (60% of the unobstructed case) by buildings and vegetation. The 18 min walk on the second path through the recreational park results in a vitamin D_3_-weighted exposure of 6.26 J for the unobstructed case or 3.94 J with obstructions (63% of the unobstructed case). The accumulated exposure after 10 min for the two routes is very similar and results in a nearly equal vitamin D_3_-weighted human exposure (see Figure 5).

**Figure 6:**
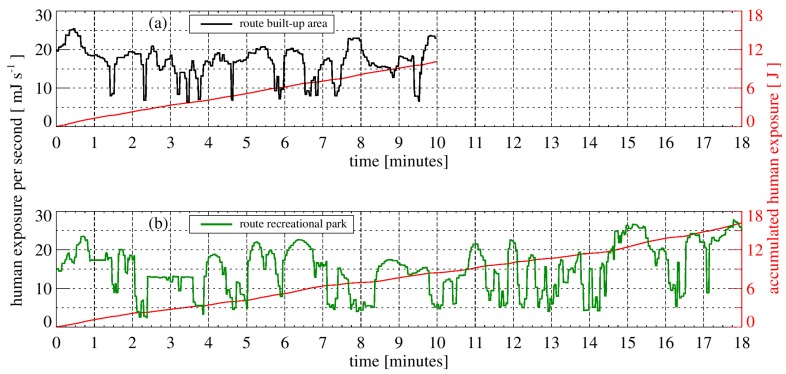
The calculated vitamin D_3_-weighted exposure of a human with summer clothing walking to the university cafeteria on the two selected routes, through a built-up area (**a**) and through a recreational park (**b**), on 21 June is shown as a function of the time. Additionally, the accumulated exposure is displayed in red.

The calculated exposure of a human with summer clothing walking to the university cafeteria on the two selected footpaths on 21 June is shown as a function of the time in Figure 6. The vitamin D_3_-weighted human exposure on the first route (“Nienburger Straße”) results in 10.14 J and the exposure on this way without obstructions would be 19.26 J (53% of the unobstructed case). The 18 min walk on the second path through the recreational park results in a vitamin D_3_-weighted human exposure of 35.44 J for the unobstructed case or 16.28 J with obstructions (46% of the unobstructed case).

## 4. Discussion

When in front of the university cafeteria, and the human face is orientated directly away from the sun, the exposure is about 20% lower (see Figure 3b). This is caused both by direct sun and diffuse sky radiation around the sun. Although the contribution of the direct beam to the exposure is small compared to the contribution of diffuse sky radiation, the direct beam contributes significantly to the variation of the exposure due to different orientations. In comparison to the environment of Figure 3b, the variation of the exposure by diffuse sky radiation can increase when the diffuse sky radiation around the sun is unobstructed, and parts of the sky opposite the sun are covered by obstructions (see Figure 3a). For a lower solar zenith angle than 52° used in Figure 3 (e.g., noon on 21 June), the absolute values are greater, but the relative shape of the functions remain nearly the same.

For the location in front of the institute on 21 March and a solar zenith angle of 75°, the direct component is only 8% of the total exposure when the human face is orientated towards the sun. The ratio of direct to diffuse exposure decreases with increasing solar zenith angle, and the diffuse component becomes the dominant factor in the variation of the exposure due to different orientations. However, the absolute exposure values for situations with greater solar zenith angles are small. Therefore, the dependence of the orientation on the exposure is of little importance in these situations.

The result of the accumulated exposure for the two investigated routes, with nearly equal values after 10 min, leads to the conclusion that vegetation in winter and spring (e.g., trees and bushes without foliage) may have a comparable impact on human exposure to buildings. However, the result of the accumulated exposure after 10 min with the obstruction information from summer showed that vegetation (e.g., trees and bushes with foliage) from the route through the recreational park has a greater impact on human exposure than buildings.

The results of this study show that, under clear sky situations, the exposure values for 21 March are still low, if only a few minutes of the lunch break are used for sun exposure. For 21 June, however, enough vitamin D_3_ can be produced with a 20 min walk during lunch break in an urban environment, if 1000 IU are assumed to lead to an adequate vitamin D status [20,27,35,36]. To directly compare our values with the results of Webb and Engelsen [37] and Engelsen [38], we performed calculations for Boston with the same clothing and similar atmospheric conditions as used in these studies. The results of these calculations agree well with the values of [37,38] for the spring and summer scenarios. However, for 21 December, the exposure time from our exposure model is lower than the exposure time from Webb and Engelsen [37]. This is probably a result of the different exposure models (plane surface model [37] and 3-dimensional human exposure model [27]), and comparisons should therefore be treated with caution.

All results are calculated for clear sky conditions. In most cases, however, there are clouds. For these conditions, the exposure is further reduced by cloudiness. The effects of clouds are complex, and usually lead to a higher spatial and temporal variation of sky radiance compared to cloudless skies [39]. An investigation of the effects of clouds would therefore be desirable, but is beyond the scope of this paper. Additionally, a varying total ozone column also results in a varying exposure. Further, it should be noted that for the calculation of the vitamin D_3_-weighted exposure, several simplifying assumptions were made, which are listed in Seckmeyer et al. [27].

Since the presented results are based on model calculations only, a validation of these calculations with measurements of the spectral radiance would be desirable. For this, a newly-developed technique may be used that measures the spectral radiance of more than 100 directions simultaneously [39]. Using this instrument, the complex and rapidly changing radiation field can be captured with a temporal resolution of seconds. However, such measurements are not yet operational, and could therefore not be used for this study. Additionally, it would be desirable to perform dosimeter measurements in an urban environment, and compare those with calculations from our exposure model. Personal dosimeters cannot replace spectroradiometer measurements, as the former often show large deviations from spectroradiometer measurements [40]. However, if dosimeters are carefully characterized and preselected, the use of dosimeters could extend the amount of data to estimate the exposure of a human in an urban environment.

## 5. Conclusions

There are large seasonal differences in the production of vitamin D that result in an insufficient vitamin D supply in humans living at middle or high latitudes. However, low UV exposure levels do not only occur in winter time; obstructions in urban environments have an impact on human exposure as well. In the current study, we demonstrated the exposure of a human in an urban environment to be dependent on the orientation of the human towards the sun. To maximize the UV exposure in an urban environment, an orientation towards the sun should be chosen when possible. Additionally, human behavior—e.g., the location and time spent outside—is the prominent factor that determines the actual UV dose received (exposure integrated over time). For humans with the goal of spending time outside to produce vitamin D_3_, a park may be more attractive and enjoyable than an urban environment. However, it was further shown that the accumulated exposure values of a moving human through a recreational park and a built-up area are about the same in winter time. This means that the impact of vegetation in winter and early spring (without foliage) on exposure is comparable to the impact of buildings. Thus, locations with as little obstructions as possible should be chosen to maximize the UV exposure and, consequently, vitamin D_3_-production. To further improve the exposure calculation of everyday situations in an urban environment, instead of simulations of clear sky conditions, actual measurements of the sky radiation for different atmospheric conditions should be considered.

## Figures and Tables

**Table 1:** Calculated vitamin D_3_-weighted exposure of a human walking to the university cafeteria on the two selected routes on 21 March and 21 June with winter and summer clothing, respectively. The values of the accumulated human exposure are given in joules (J) and international units (IU). The conversion to IU can be performed by multiplying the human exposure in J by the conversion factor of 70.97 (IU J^−1^), as stated in [27].

Route	Exposure of a Human 21 March (Winter Clothing)		Exposure of a Human 21 June (Summer Clothing)	
	(J)	(IU)	(J)	(IU)
Built-up area (unobstructed)	3.48	247	19.26	1367
Built-up area (obstructed)	2.10	149	10.14	720
Recreational park (unobstructed)	6.26	444	35.44	2515
Recreational park (obstructed)	3.94	280	16.28	1155
